# Tumor microenvironment participates in metastasis of pancreatic cancer

**DOI:** 10.1186/s12943-018-0858-1

**Published:** 2018-07-30

**Authors:** Bo Ren, Ming Cui, Gang Yang, Huanyu Wang, Mengyu Feng, Lei You, Yupei Zhao

**Affiliations:** Department of General Surgery, Chinese Academy of Medical Sciences, Peking Union Medical College, Peking Union Medical College Hospital, Beijing, 100023 People’s Republic of China

**Keywords:** Pancreatic cancer, Tumor microenvironment, Desmoplasia, Immunosuppression, Metastasis

## Abstract

Pancreatic cancer is a deadly disease with high mortality due to difficulties in its early diagnosis and metastasis. The tumor microenvironment induced by interactions between pancreatic epithelial/cancer cells and stromal cells is critical for pancreatic cancer progression and has been implicated in the failure of chemotherapy, radiation therapy and immunotherapy. Microenvironment formation requires interactions between pancreatic cancer cells and stromal cells. Components of the pancreatic cancer microenvironment that contribute to desmoplasia and immunosuppression are associated with poor patient prognosis. These components can facilitate desmoplasia and immunosuppression in primary and metastatic sites or can promote metastasis by stimulating angiogenesis/lymphangiogenesis, epithelial-mesenchymal transition, invasion/migration, and pre-metastatic niche formation. Some molecules participate in both microenvironment formation and metastasis. In this review, we focus on the mechanisms of pancreatic cancer microenvironment formation and discuss how the pancreatic cancer microenvironment participates in metastasis, representing a potential target for combination therapy to enhance overall survival.

## Background

Despite the significant advances in cancer research, pancreatic cancer is still a deadly disease. According to the latest epidemiological data, a total of 55,440 patients were newly diagnosed with pancreatic cancer, and 44,330 people died from the disease in the United States. In contrast to other cancer types with continuous increases in survival, that of pancreatic cancer decreased slightly, and the disease is commonly diagnosed at an advanced stage, leading to a 5-year survival rate of only 8% [1]. Among patients with pancreatic cancer, 90% carry a *KRAS* mutation, which is considered a driver gene for pancreatic cancer progression, and 50–80% have inactivating mutations in *TP53*, *CDKN2A*, and *SMAD4* [2]. Pathological results have shown that the most common type of pancreatic cancer is pancreatic ductal adenocarcinoma (PDAC). Pancreatic cancer is associated with an extremely poor prognosis and high mortality because it is usually diagnosed at advanced stages with metastasized pancreatic cancer cells, requiring cellular elements that contribute to pancreatic cancer microenvironment formation.

Increasing interest has been focused on the tumor microenvironment of pancreatic cancer. The pancreatic cancer microenvironment consists of cancer cells, stromal cells and extracellular components. Stromal cells that contribute to pancreatic cancer progression are mainly pancreatic stellate cells (PSCs), regulatory T cells (Tregs), myeloid-derived suppressor cells (MDSCs), and tumor-associated macrophages (TAMs). These cells and cancer cells can secrete extracellular components, such as extracellular matrix (ECM), matrix metalloproteinase (MMP), growth factors, and transforming growth factor-β (TGFβ), to maintain the microenvironment. Recent studies have demonstrated that the pancreatic cancer microenvironment plays a critical role in PDAC progression [3], revealing the relationship between the microenvironment and metastasis. There are two major characteristics of the pancreatic cancer microenvironment: dense desmoplasia and extensive immunosuppression [4]. These two features can facilitate pancreatic cancer cell proliferation, the evasion of immune surveillance via the direct inhibition of anti-tumor immunity or the induction of immunosuppressive cell proliferation and metastasis. Therefore, this review discusses pancreatic cancer microenvironment formation and the mechanisms by which the microenvironment participates in metastasis to elucidate the relationship between the microenvironment and metastasis.

### Characteristics of the pancreatic cancer microenvironment

#### Desmoplasia

In pancreatic cancer, there is extensive fibrosis at primary tumor sites, which is termed desmoplasia and documented in the pathology of PDAC. The clinical manifestations of desmoplasia are overexpression of ECM proteins and extensive transformation of fibroblastic-type cells to a myofibroblastic phenotype [5]. Desmoplasia is associated with a poor prognosis by promoting the progression of pancreatic cancer and resistance to chemotherapy [6]. These cellular components can promote desmoplasia in the pancreatic cancer microenvironment through the secretion of certain molecules, such as TGFβ, fibroblast growth factor 2 (FGF2), and connective tissue growth factor (CTGF). Among these factors, TGFβ is notable for its dual nature in cancer. It can prevent neoplastic cell growth during pancreatic intraepithelial neoplasm-1 (PanIN-1) and PanIN-2 while promoting growth during PanIN-3 due to the loss of *SMAD4* and the canonical arm of the TGFβ pathway [7, 8], and it interacts at many levels with the RAS-RAF-ERK pathway [9]. Specimens from patients have shown that ECM deposition in primary tumors is associated with a poor prognosis of patients with pancreatic cancer [10]. Desmoplasia can establish a hypoxic microenvironment by enhancing the functions of antiangiogenic factors. Hypoxia, which is caused by an insufficient vasculature, is important for pancreatic cancer aggressiveness, including metabolic reprogramming, inhibition of apoptosis, sustained proliferation, treatment resistance, invasion and metastasis [11]. In contrast to other solid tumors, pancreatic cancer cells can secrete antiangiogenic factors, such as angiostatin, endostatin, and pigment epithelium-derived factors, into the hypovascular microenvironment, and ECM deposition can amplify endostatin production by cancer cells to enhance hypoxia [12–14]. Recent studies have reported that desmoplasia components might be potential therapeutic targets of pancreatic cancer. For instance, hyaluronan degradation by hyaluronidase PEGPH20 has been shown to increase vessel patency, drug delivery and survival in preclinical trials [15, 16], and the efficacy of hyaluronidase PEGPH20 plus gemcitabine is currently being evaluated in an ongoing phase I/II trial (NCT01453153).

#### Immunosuppression

The relationship between anti-tumor immunity and pancreatic cancer progression has been a hot topic in studies of pancreatic cancer. The immune system during pancreatic cancer can not only suppress tumor development or progression by destroying cells with mutations and prevent them from developing into tumor cells, but it can also promote pancreatic cancer progression by establishing favorable conditions for immunosuppression and metastasis [17, 18]. The tumor infiltrating lymphocyte (TIL) population in pancreatic cancer shows clinical correlates that higher proportions of CD4^+^, CD8^+^ and dendritic cells (DCs) of TILs can improve the prognosis of affected patients with [19]. Among these immune cells, CD8^+^ T cells play an essential role in killing tumor cells, and a greater number of cancer cell adjacent cytotoxic T cells significantly correlates with survival [20]. Normally, antigen-presenting cells (APCs), such as macrophages and DCs, process tumor antigens for display on major histocompatibility complex (MHC) I molecules, which activate subsequent CD8^+^ T cells to kill tumor cells via the granzyme, perforin, and first apoptosis signal (Fas)/FasL pathway.

Pancreatic cancer cells themselves are critical for immunosuppression by inhibiting CD8^+^ T cell activation and upregulating the existing regulatory immune cells. Pancreatic cancer has been shown to decrease its MHC I expression to prevent recognition by CD8^+^ T cells for evasion [21]. Furthermore, Fas expression is downregulated in pancreatic cancer cells, which leads to resistance to Fas-mediated apoptosis, and pancreatic cancer can induce apoptosis of CD8^+^ T cells by Fas/FasL counterattack [22]. Kaplan-Meier survival analysis demonstrated that high levels of Fas cytoplasmic expression in pancreatic cancer cells are significantly associated with a better outcome of pancreatic cancer [23]. Immunosuppressive cytokines such as interleukin (IL)-10 and TGFβ are also secreted during pancreatic cancer to help fibrosis, immunosuppressive phenotype formation and recruit cells involved in immune evasion to overcome the anti-tumor immunity [24–26]. Indoleamine 2,3-dioxygenase (IDO) catalyzes the conversion from tryptophan to kynurenine, which leads to anergy of anti-tumor T cells and enhances Treg function [27–29]. Moreover, high expression of IDO in pancreatic cancer cells can mediate nature killer (NK) cells dysfunction [30]. Commonly, Forkheadbox protein 3 (Foxp3), a transcription factor considered a marker of Tregs, is also expressed on cancer cells. Cancer-Foxp3 causes immunosuppression by inducing Treg accumulation via CCL5 and negatively correlates with a poor prognosis in pancreatic cancer [31]. Finally, pancreatic cancer cells express cytotoxic-T-lymphocyte associated protein 4 (CTLA-4) and the ligand for programmed cell death protein-1 (PD-1), PD-L1 [32], which is activated by the EGFR/MAPK pathway from myeloid cells [33], inhibiting T cell function. Currently, CTLA-4 and PD-1/PD-L1 have been established as therapeutic targets [34, 35]. These molecules can be blocked by monoclonal antibodies, including ipilimumab, nivolumab, and pembrolizumab. However, immune-checkpoint inhibition monotherapy may not be effective in pancreatic cancer, potentially because of the low PD-L1 expression in pancreatic cancer, highly complicated interaction between the tumor and stroma, and desmoplasia [34]. Therefore, immunotherapies combined with other therapies, such as surgery, chemotherapy, radiotherapy, targeted therapy, and other immunotherapies (Table 1), may overcome the resistance of immunotherapy

**Table 1:** Recent clinical trials concerning immune-checkpoint inhibitors in pancreatic cancer

NCT Number	Status	Phase	Tumor types	Interventions	Monotherapy/Combination
NCT02305186	Recruiting	Phase 1/2	Pancreatic Cancer	Pembrolizumab + Neoadjuvant chemoradiation	Combination
NCT02930902	Recruiting	Phase 1	Pancreatic Cancer	Pembrolizumab + Paricalcitol + Surgical Resection or Pembrolizumab + Paricalcitol + Surgical Resection + Gemcitabine + Nab-pacilitaxel	Combination
NCT02451982	Recruiting	Phase 1/2	Pancreatic Cancer	Cyclophosphamide + GVAX pancreatic cancer or Cyclophosphamide + GVAX pancreatic cancer + Nivolumab	Combination
NCT02866383	Recruiting	Phase 2	Metastatic Pancreatic Cancer	Nivolumab + Ipilimumab + Radiotherapy	Combination
NCT03519308	Recruiting	Early Phase 1	Pancreatic Cancer	Nivolumab + Nab-Paclitaxel + Gemcitabine + Paricalcitol	Combination
NCT03404960	Recruiting	Phase 1/2	Pancreatic Cancer	Niraparib + Nivolumab or Niraparib + Ipilimumab	Combination
NCT03104439	Recruiting	Phase 2	Microsatellite Stable Colorectal Cancer Pancreatic Cancer MSI High Colorectal Cancer	Nivolumab + Ipilimumab + Radiation therapy	Combination
NCT01473940	Active, not recruiting	Phase 1	Ductal Cell Adenocarcinoma of the Pancreas Recurrent Pancreatic Cancer Stage III Pancreatic Cancer Stage IV Pancreatic Cancer	Ipilimuma + Gemcitabine hydrochloride	Combination
NCT01896869	Suspended	Phase 2	Metastatic Pancreatic Adenocarcinoma	Ipilimumab + Vaccine + FOLFIRINOX	Combination

### Pancreatic cancer interacts with stromal cells in the microenvironment

The pancreatic cancer microenvironment is characterized as dense desmoplasia and extensive immunosuppression with abundant cellular elements, mainly involving pancreatic cancer cells, Tregs, MDSCs, TAMs, and PSCs. Pancreatic cancer cells can activate or recruit other cellular elements for desmoplasia and immunosuppression and finally facilitate metastasis. Additionally, these cells can promote pancreatic cancer cell growth, proliferation, and maintenance of stemness. Compared with other types of cancer, pancreatic cancer cells only consist of approximately 10–30% of the cellular components, while dense stroma makes up 80% of the tumor mass in some patients [36]. Thus, other tumor-supporting cells are critical for the pancreatic cancer microenvironment to facilitate desmoplasia, immunosuppression, and pancreatic cancer progression.

#### PSCs

PSCs are considered myofibroblast-like cells that are located in exocrine regions of the pancreas [37] and share similarities with hepatic stellate cells. Quiescent PSCs store vitamin A and produce matrix metalloproteinases (MMPs) such as MMP-2, MMP-9, and MMP-13 and their inhibitors for turnover of the extracellular matrix (ECM), to maintain the modeling of normal tissue [38].

During PDAC, quiescent PSCs are activated by a variety of factors, such as IL-1, IL-6, hypoxia inducible factor 1α (HIF1α), and TGFβ, to transform them into the myofibroblast-like phenotype [39]. Activated PSCs are classified as a loss of cytoplastic lipid droplets, upregulation of MMP and ECM proteins [37], and activated PSCs acquire proliferative capacity. Additionally, activated PSCs play a vital role in the pancreatic cancer microenvironment by secreting molecules such as TGFβ, IL-6, stromal cell-derived factor-1 (SDF-1), hepatocyte growth factor (HGF) and galectin-1 to promote pancreatic cancer progression [40].

PSCs are cells that mainly contribute to desmoplasia in pancreatic cancer. Recent evidence has demonstrated that PSCs can induce desmoplasia via numerous signaling pathways, such as IL-6, paracrine sonic hedgehog (SHH) signaling, the vitamin D receptor (VDR) pathway, and the CXCL12/CXCR4 pathway [41]. Among these signaling pathways activating PSCs, SHH expression in pancreatic cancer cells is induced by KRAS by the activation of nuclear factor-κB (NFκB) [42]. GLI1, the target gene of SHH, shows a predominant signal in the stromal compartment [43]. Thus, pancreatic cancer cells express SHH to activate GLI1 in stroma to create a tumor-supportive microenvironment. In addition, PSC inhibition is a potential therapeutic target because it can inhibit desmoplasia [3, 44].

Recently, the relationship between immunosuppression and PSCs has been demonstrated. CXC chemokine ligand 12 (CXCL12) secreted by PSCs has the ability to reduce the migration of CD8^+^ T cells into the peritumoral stroma of pancreatic cancer [45]. Galectin-1 promotes immunosuppression in the pancreatic cancer microenvironment by inducing T cell apoptosis and Th2 cytokine secretion [46]. In addition to suppressing T cells directly, PSCs can recruit other immune cells into the tumor microenvironment to assist their immunosuppressive function. For instance, PSCs recruit Tregs via the IP-10/CXCL10 pathway [47], and IP-10 is elevated in pancreatic cancer patients, which is associated with a high stroma content and a decreased median overall survival [48]. Moreover, differentiation from peripheral blood monocytes into MDSCs can be induced by PSCs via the IL-6/signal transducer and activator of transcription 3 (STAT3) pathway to suppress T cell proliferation [49]. Although studies of the relationship between PSCs and immune evasion are still in the early stage, PSCs have potential as a target of pancreatic cancer to enhance immunotherapy in the future.

#### Tregs

CD4^+^ CD25^+^ Tregs (hereafter referred to as Tregs), also known as suppressor T cells, are a subtype of T cells that maintain tolerance to self-antigens and prevents autoimmune disease by suppressing or downregulating the induction and proliferation of effector T cells [50]. They are recruited by pancreatic cancer cells into the tumor microenvironment and play an important role in immunosuppression during pancreatic cancer progression. Higher proportions of Tregs in TILs are associated with progression and a poorer prognosis of patients with pancreatic ductal adenocarcinoma (PDAC) [51]. Foxp3 and CTLA-4 mRNA expression are higher in Tregs from the peripheral blood of patients with progressed and advanced pancreatic cancer, and there should further be a positive correlation between the IL-10 or TGFβ levels and the progression of pancreatic cancer [52].

Tregs can suppress tumor immunity through a variety of pathways. For instance, Tregs secrete suppressive cytokines and molecules, such as IL-10 and TGFβ, consistent with clinical findings, to inhibit effector T cell functions [53]. Another mechanism by which Tregs induce effector T cell cytolysis involves granzyme B [54, 55], the TRAIL pathway [56] and galectin-1 [57]. Moreover, Tregs can promote pancreatic cancer growth via the TRAIL pathway [58]. In addition, Tregs bind to IL-2 competitively to starve effector cells [59]. CTLA-4 expressed by Tregs can upregulate the IDO pathway in DCs [60] and effector T cells [29] and lead to their dysfunction.

#### MDSCs

MDSCs, which are defined as a heterogeneous population of immature myeloid cells in spleen and tumor, play a critical role in immunosuppression of pancreatic cancer. The markers of MDSCs are CD11b^+^ CD33^+^ HLA-DR^−^ in humans. Patients with pancreatic cancer have higher MDSC and pro-MDSC cytokine levels in the peripheral blood, and MDSCs in peripheral blood may be a predictive biomarker of chemotherapy failure in pancreatic cancer patients [61]. Pancreatic cancer consistently induces the proliferation and mobility of MDSCs within the bone marrow to the tumor microenvironment [62] via cytokines, especially granulocyte macrophage colony-stimulating factor (GM-CSF), which is widely studied and produced by pancreatic cancer cells. GM-CSF is associated with differentiation from myeloid progenitor cells to MDSCs and MDSC recruitment to the pancreatic cancer microenvironment [63]. The upregulation of GM-CSF is induced by the driver gene *KRAS*^*G12D*^ [64], which is mutated in more than 90% of pancreatic cancer cases [2].

When MDSCs enter the pancreatic cancer microenvironment, they can suppress the functions of effector T cells via numerous pathways. For instance, MDSCs release reactive oxygen species (ROS), induced by cytokines such as TGFβ and IL-10 [65] released from other cells, causing oxidative stress in T cells. As a result, the CD3ζ chain in T cells cannot be translated, which inhibits antigen-dependent proliferation [66]. Polymorphonuclear cells (PMN-MDSC) possess the ability to take up, process and present antigens on MHC I proteins. These complexes can present antigen to CD8^+^ T cells to induce immune tolerance for immune evasion [67]. Arginase 1 (Arg1), depending on STAT3 signaling [68], and inducible nitric oxide synthase (iNOS) in MDSC are capable of depleting L-arginine from the tumor microenvironment, such that T cells are unable to multiply. STAT3 signaling pathways can be activated in MDSCs by IL-10, and STAT3 phosphorylation not only induces Arg1 expression but also upregulates PD-L1 expression on the surface of MDSCs [69], which suppresses T cell activation. In addition, MDSCs in vitro have been shown to be capable of inducing Treg development, the functions of which have been previously discussed, and targeted depletion of an MDSC subset—Gr-MDSC—leads to the accumulation of activated CD8^+^ T cells, apoptosis of tumor cells, and remodeling of the tumor stroma [70].

#### TAMs

Furthermore, macrophages derived from monocytes are phagocytic cells involved in the innate immune system. They participate in desmoplasia and immunosuppression during pancreatic cancer progression. A recent study showed that mutant *KRAS*^*G12D*^ can upregulate intercellular adhesion molecular-1 (ICAM-1) in acinar cells, serving as a chemoattractant for macrophages [71]. As a result of their plasticity, macrophages consist of a heterogeneous population of cells with different functional and phenotypic characteristics [72]. According to the activation mechanism, macrophages are classified as M1 (activated by IFN-γ and TLR ligands, the expression of higher levels of IL-12, IL-23, MHC II, and inducible nitric oxide synthase, and tumoricidal) or M2 (activated by IL-4 and IL-13, the expression of higher levels of IL-10 and TGFβ, and facilitate tumor progression) [73, 74]. Flow cytometry has demonstrated that tumor-derived factors stimulate the differentiation of macrophages, with mixed M1-like and M2-like phenotype polarization [75]. The M2-like phenotype of tumor-associated macrophages (TAMs) is immunosuppressive, and overall survival is shorter in pancreatic cancer patients with high-density M2 macrophage than low-density M2 macrophage infiltration [76]. Factors in the pancreatic cancer microenvironment, such as CSF-1, IL-4, IL-13, TGFβ and IL-10, can promote myeloid progenitor cell differentiation into monocytes and macrophages and recruit them to the tumor microenvironment [73, 77–79].

An immunosuppressive activity of TAMs includes the secretion of immunosuppressive cytokines, chemokines, and enzymes, such as TGFβ, IL-10, CCL17, and CCL22 [80]. Similar to MDSCs, TAMs can express more Arg1 to interfere with the metabolism of effector T cells [81], and factors such as TGFβ, IL-10, and prostaglandin E2 (PGE2) released by TAMs favor Treg recruitment and inhibit CD8^+^ T cells activities [82]. Moreover, NLRP3 signaling in macrophages drives the differentiation of CD4^+^ T cells into tumor-promoting T helper type 2 cells (Th2 cells), Th17 cells, and the Treg population, while suppressing Th1 cell polarization and cytotoxic CD8^+^ T cell activation [83]. TAMs may also induce apoptosis of T cells by expressing PD-L1 on their surface, which is similar to pancreatic cancer cells and MDSCs. Additionally, dectin-1 activation on macrophages via galectin-9 in the tumor microenvironment results in peritumoral immune tolerance in pancreatic cancer [84].

Furthermore, TAMs contribute to desmoplasia by facilitating PSCs. For instance, M2 macrophages can promote pancreatic fibrosis [85], and macrophages can drive PDAC fibrosis, immunosuppression and metastasis via the PI3Kγ pathway [86]. In vitro co-culture of macrophages and PSCs has demonstrated that the macrophage-stellate cell interaction is a pivotal component of desmoplasia in PDAC [87], and previous studies have demonstrated that TAMs can upregulate PSC functions. For example, TAMs can stimulate PSC proliferation and ECM secretion via TGFβ1 and PDGF, respectively [88]. In addition, hypoxia can lead to the recruitment of macrophages to activate PSCs through CCL2 secretion induced by HIF1, enhancing desmoplasia by PSCs [89]. Overall, the functions of TAMs seem to be diverse because they participate in many steps of pancreatic cancer progression.

#### Pancreatic cancer stem cells

Pancreatic cancer stem cells (CSCs) are considered a small subset of pancreatic cancer cells that can self-renew and generate the heterogeneous lineages of cancer cells in the tumor. They are a fundamental driving force of pancreatic cancer initiation and progression [90]. Recent studies have demonstrated that pancreatic CSCs can model the tumor microenvironment to favor their stemness maintenance, including self-renewal, tumorigenic, and metastatic potential. The main signaling pathways involved in this process are Wnt/β-catenin, hedgehog, notch, NF-κB, PI3K/Akt and PTEN, and they are dysregulated in pancreatic cancer [91–95].

Pancreatic CSCs can differentiate into bulk tumor cells partially in response to autocrine growth factor signaling. For example, activin and nodal, secreted by pancreatic CSCs, can cause pancreatic cancer cells to form spheres by binding to the receptors Alk4/7 [96]. Xenograft tumors from pancreatic CSCs with decreased levels of Alk4/7 can enhance sensitivity to gemcitabine and lead to longer survival times than pancreatic CSCs with high levels of Alk4/7 [96].

**Fig. 1:**
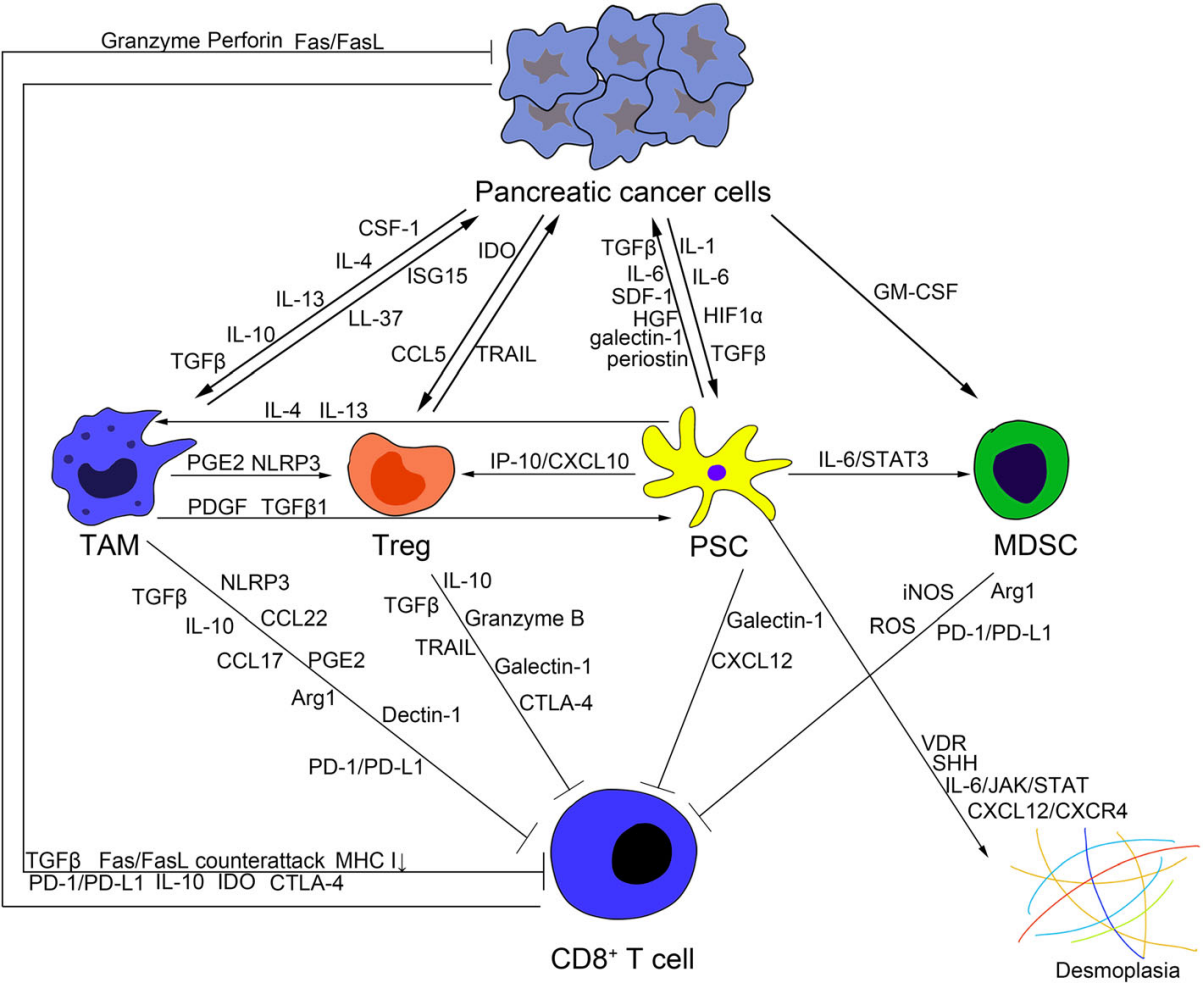
Development of the pancreatic cancer microenvironment. Pancreatic cancer cells secrete cytokines and chemokines to recruit or activate stromal cells for desmoplasia and immune evasion, including myeloid-derived suppressor cells (MDSCs), tumor-associated macrophages (TAMs), regulatory T cells (Tregs), and pancreatic stellate cells (PSCs). Among them, PSCs are the main source of ECM deposition, and the TAM-PSC axis can facilitate desmoplasia. These cells within the pancreatic cancer microenvironment can help pancreatic cancer cells inhibit CD8^+^ T cells to overcome the immune surveillance by expressing or producing various factors, such as IL-10, TGFβ, PD-L1, and IDO

In addition to pancreatic CSCs themselves, stromal cells can maintain the pancreatic CSC population via paracrine signaling pathways. PSCs can secrete activin and nodal, similar to pancreatic CSCs, to promote the formation of tumor spheres in vitro and invasiveness of pancreatic CSCs in an Alk4-dependent manner [97]. HGF from PSCs can promote self-renewal of c-Met^High^ pancreatic CSCs. In addition to the observations for PSCs, TAMs can also secrete factors to maintain the functions of pancreatic CSCs. A clinical study has demonstrated a positive correlation between the expression of CD44/CD133 and CD204, a marker of TAMs, and higher expression of these 3 markers was associated with shorter overall survival and disease-free survival [98]. Pancreatic CSCs can secrete IFNβ to stimulate TAM production of IFN-stimulated factor ISG15 to enhance the CSC phenotype in vitro and in vivo [99]. TAMs also produce leucine leucine (LL)-37 in the response to tumor growth factor, associated with a shorter overall survival, to increase pluripotency-associated gene expression, self-renewal, invasion and tumorigenicity of pancreatic CSCs via formyl peptide receptor 2 (FPR2)- and P2X purinoceptor 7 receptor (P2X7R)-dependent mechanisms [100]. In conclusion, the pancreatic cancer microenvironment can influence the stemness of pancreatic CSCs by multiple pathways (Fig. 1).

### The pancreatic cancer microenvironment participates in metastasis

Metastasis is a major cause of morbidity and mortality in patients with pancreatic cancer, and the main pathways of metastasis are local invasion and lymphatic metastasis. Currently, only 20% of patients have resectable pancreatic cancer, with an 80% relapse rate. The majority of pancreatic cancer patients are locally advanced and unresectable disease due to vascular involvement or widespread metastasis, and the main pathways of metastasis are local invasion and lymphatic metastasis. Most patients will die with metastases to the liver, lung and/or peritoneum, the most common sites of distal metastasis [101]. Steps of successful metastasis include angiogenesis/lymphangiogenesis, epithelial-mesenchymal transition (EMT), invasion to surround tissues and migration, formation of a pre-metastatic niche, and growth at the metastatic site. Cancer cells should be depleted by anti-tumor immune cells, especially CD8^+^ T cells and NK cells, during each step of metastasis. Unfortunately, pancreatic cancer cells can overcome the anti-tumor immunity and metastasize to other sites due to the recruited immunosuppressive cells and their interactions.

#### Angiogenesis

Pancreatic cancer cells and other recruited immunosuppressive cells within the tumor microenvironment, such as TAMs and MDSCs, induce angiogenesis for the tumor blood supply and metastasis by secreting several pro-angiogenic factors, cytokines, and growth factors. Among these molecules, vascular endothelial growth factor (VEGF) plays a central role in the angiogenesis of pancreatic cancer, and the secretion of VEGF is regulated by multiple signaling pathways. For pancreatic cancer cells, STAT3 is constitutively expressed in pancreatic cancer cells, which activates the expression of VEGF for angiogenesis [102]. Mucin (MUC) 1 in pancreatic cancer cells can induce hypoxia for VEGF-A and platelet-derived growth factor (PDGF) B production, which contributes to endothelial cell tube formation [103]. NF-κB is another protein that regulates VEGF expression. Activated NF-κB in pancreatic cancer cells can upregulate VEGF [104], and xanthohumol can decrease VEGF expression and inhibit angiogenesis of pancreatic cancer by suppressing the NF-κB pathway [105]. TAMs are also involved in angiogenesis through the secretion of VEGF [106], and pharmacological targeting of tumor-infiltrating macrophages is associated with impaired angiogenesis [107]. In addition to VEGF, a recent study of xenograft models has shown that the angiogenesis of TAMs can be induced by pancreatic cancer cells, which secrete IL-35 to recruit TAMs and activate their CXCL1 and CXCL8 to stimulate angiogenesis, and the combination of a neutralizing antibody against IL-35 and gemcitabine significantly decreased monocyte infiltration and microvessel density [108].

#### Lymphangiogenesis and lymphatic metastasis

Similar to angiogenesis in many regards, tumor-associated lymphangiogenesis is a key step for pancreatic cancer progression, especially lymph node metastasis of pancreatic cancer. New lymphatic vessel growth can be directed by many factors derived from pancreatic cancer cells and other cells, such as M2-like TAMs [109], in the tumor microenvironment. Clinical results have shown that a high lymphatic vessel density in pancreatic ductal adenocarcinoma (PDAC) correlates with the tumor differentiation status, increased lymph node metastasis and decreased overall survival [110–113]. In comparison to normal lymph nodes, more Tregs, MDSCs, immature and tolerogenic DCs and immunosuppressive cytokines [114, 115] lie in tumor-draining lymph nodes, and the Treg density in pancreatic cancer tissue and lymph nodes correlates with pancreatic cancer lymphatic metastasis [116]. Coincidentally, a greater quantity of CD10^+^ PSCs in PDAC tissue indicates positive nodal metastasis and a shorter survival time [117], and M2-like TAMs can increase the lymphatic vessel density in pancreatic cancer and accelerate lymphatic metastasis [118]. These results indicate that these cells may be involved in lymphangiogenesis and lymphatic metastasis. Molecular components involved in lymphangiogenesis resemble those in angiogenesis, and VEGF-C/D appears to be important for lymphangiogenesis. Clinical studies have shown that VEGF-C/D expression increases in PDAC patients and correlates with increased lymphatic vessel invasion, lymph node metastasis, and a decreased five-year survival [119]. Knockdown of VEGF-C by anti-VEGF-C shRNA can decrease the lymphatic vessel density and inhibit tumor growth [120]. A recent study has demonstrated that microRNAs are involved in the regulation of VEGF-C production in PDAC cells. MicroRNA-206 can block K-Ras and annexin-A2 gene expression to suppress PDAC progression and downregulate VEGF-C production to inhibit lymphatic vessel formation through an NF-κB-independent pathway. In addition to VEGF, other factors within the pancreatic cancer microenvironment also participate in lymphangiogenesis and lymphatic metastasis. Inhibition of pancreatic cancer-derived SHH, an important molecule in embryonic development, reduces lymphangiogenesis and lymph node metastasis in a pancreatic cancer mouse model [121]. The proline TP53 variant stimulates lymphangiogenesis in the orthotopic pancreatic cancer mouse model [122].

#### Epithelial-mesenchymal transition

EMT in cancer is a process by which the cancer cells lose their cell-cell adhesion capacity and break through the basement membrane for invasion, during the initiation of metastasis. Specimens from pancreatic cancer patients indicate that EMT status is associated with portal vein invasion and lymph node metastasis [123]. A tag and track pancreatic epithelial cell experiment in premalignant lesions and pancreatic cancer mouse models has shown that pancreatic epithelial cells can invade and enter the bloodstream to become circulating epithelial cells (CECs), maintaining the mesenchymal phenotype even at the PanIN stage, and inflammation enhances EMT, invasiveness, and dissemination of pancreatic epithelial cells [124]. Another premalignant lesion, the intraductal papillary mucinous neoplasm (IPMN), which is classified as low-grade dysplasia (adenoma), intermediate-grade dysplasia (borderline), and high-grade dysplasia (carcinoma in situ) [125] and can be detected at an early stage, also undergoes EMT. Franses et al. demonstrated that CECs can be detected in mice and 88% of patients with IPMNs, and RNA-seq analysis showed that EMT of pancreatic epithelial cells in IPMNs may be driven by MUC genes [126]. Cellular elements in the tumor microenvironment, such as TAMs and PSCs, which provides the links among inflammation, premalignant lesions and cancer, can facilitate EMT. For example, M2-like TAMs can increase MMP2 and MMP9 activity in pancreatic cancer cells and decrease E-cadherin, indicating that EMT and Toll-like receptor (TLR) 4 expression and IL-10 production, are upregulated in M2-like TAMs to stimulate EMT when cocultured with pancreatic cancer cells [127]. Galectin-1, a key protein in immunosuppression and secreted by PSCs, promotes the development and metastasis of PDAC. Overexpression of galectin-1 in PSCs strongly correlates with increased expression of EMT markers in both the orthotopic xenograft tumor and in metastatic lesions of naked mice [128]. The IL-6/STAT3 pathway in PSCs, which recruits MDSCs to the pancreatic cancer microenvironment, also displays the function of promoting EMT by activating nuclear factor erythroid 2 (Nrf2) [129]. In addition to TAMs and PSCs, the relationship between pancreatic CSCs and EMT cancer cells may promote metastasis due to similar molecular characteristics, treatment resistance, and the capacity for invasion [130]. However, the exact association with EMT and pancreatic CSCs must be further investigated.

#### Invasion and migration

Invasion and migration are important for pancreatic cancer metastasis, especially hematological dissemination. Once pancreatic cancer cells invade capillaries in the tumor tissue, they can enter portal veins for distal metastasis, such as metastasis to the liver and lung. The pancreatic cancer microenvironment can facilitate metastasis by promoting pancreatic cancer cells invasion and migration. For instance, TAMs promote invasion by secreting matrix proteins and proteases to alter the ECM composition. Macrophage inflammatory protein-3α (MIP-3α) derived from TAMs in human pancreatic cancer tissue is considered a regulator of pancreatic cancer cell invasion [131]. MIP-3α can bind to CCR6 on pancreatic cells to upregulate their MMP9 expression, which increases pancreatic cancer cells invasion in collagen IV [132, 133]. Cancer-associated fibroblasts (CAFs) express high levels of palladin, an actin-associated protein, which promotes pancreatic cancer cells invasion by remodeling the ECM by regulating the activity of the small GTPase Cdc42 [134]. Among these cells in the microenvironment, PSCs play a more important role in tumor invasion and migration. In vitro studies have shown that CD10^+^ PSCs can promote the invasiveness of SUIT-2 pancreatic cancer cell lines in a murine cotransplantation model [117], and collagen-I, produced by PSCs, is the major mediator of PSC-induced haptokinesis of Panc1 and haptotaxis of UlaPaCa by activating FAK signaling via binding to integrin α2β1 [135]. Hypoxia plays a critical role in pancreatic cancer progression by inducing HIF1 to activate numerous genes involved in invasion and metastasis, such as NF-κB [136], MMP-2 [137, 138], quiescin-sulfhydryl-oxidase-1 (QSOX1) [139], CX3CR1 [140], and lysyl-oxidase (LOX) [141]. Clinical specimens have shown that SHH, induced by hypoxia via a ligand-independent mechanism [142], is overexpressed in pancreatic cancer cells and activates PSCs to secrete high levels of perineural invasion-associated molecules to promote perineural invasion in pancreatic cancer [143]. Galectin-1, which has been discussed previously, is expressed by PSCs and can induce PSC secretion of SDF-1 by NF-κB signaling and increase the migration and invasion of pancreatic cancer cells [144]. HGF, secreted by PSCs, can bind to c-Met on the surface of pancreatic cancer cells to promote their invasion and migration via the HGF/c-Met/survivin pathway [145], which is negatively regulated by the p53/p21 pathway, and HGF inhibition by AMG102 antibody can reduce pancreatic cancer metastasis dramatically in an orthotopic model of pancreatic cancer and the pancreatic cancer cell line AsPC-1 [146]. Periostin, which is a 90-kD secretory protein that was originally identified as an osteoblast-specific factor, is aberrantly upregulated in PSCs [147]. Abnormally high expression levels of periostin can increase α-SMA, periostin, collagen-1, fibronectin and TGFβ expression in PSCs and can promote growth, resistance to starvation, and invasion of pancreatic cancer cells [147]. Further studies have demonstrated that periostin creates the tumor-supportive microenvironment by binding to EGFR to trigger the Akt and Erk pathway [148].

#### Pre-metastatic niche

The entry of circulating tumor cells (CTCs), considered the “seed” of metastasis, into secondary or distant organ sites and growth at metastatic sites are affected by the local microenvironment encountered by CTCs. Primary tumors can prepare the supportive microenvironment, or “soil”, in metastatic sites, termed the pre-metastatic niche [149]. Molecular and cellular components, containing exosomes and factors secreted by the tumor, stroma, and bone-marrow-derived cells, can alter the pre-metastatic niche for pancreatic cancer colonization. The pre-metastatic niche can not only promote pancreatic cancer progression directly but also induce tumor dormancy at metastatic sites for recurrence at metastatic sites.

Liver is the main destination for pancreatic cancer distal metastasis, and factors derived from the pancreatic primary tumor microenvironment can induce fibrosis in liver to form a pre-metastatic niche by hepatic stellate cells (HSCs) that is similar to PSCs. For instance, a coculture study has shown that pancreatic cancer cells can directly stimulate HSC proliferation and matrix synthesis, including collagen I and c-fibronectin protein, and form an immunosuppressive microenvironment. These effects can be inhibited by antibodies against fibroblast growth factor 2 (FGF2), TGFβ1, and PDGF [150]. Similar to the occurrence of EMT at premalignant lesions, factors derived from premalignant lesions can also activate HSCs. Grunwald et al. [151] found that pancreatic tissue from patients with chronic pancreatitis, PanIN, and PDAC expresses higher levels of tissue inhibitor of metalloproteinases-1 (TIMP1) than normal pancreas. The premalignant lesions in KPC mice express TIMP1 and secrete it into the circulation to activate HSCs by binding to CD63 to trigger the PI3K pathway, but not TIMP1 protease inhibitor activity. Moreover, TIMP1 can increase the susceptibility of liver to pancreatic cancer cells. Subsequently, studies examining mutant p53 in pancreatic cancer have indicated that mutant p53 cannot inhibit pancreatic cancer progression but correlates with lymph node metastasis and upregulated PDGF receptor β signaling, which stimulate pancreatic cancer cell invasion in vitro and metastasis formation in vivo. Furthermore, the expression of PDGF receptor β correlates with poor disease-free survival in pancreatic cancer patients [152, 153]. In addition, mutant p53 together with TGFβ in pancreatic cancer cells can increase its ability to colonize the portal vein and hepatic sinusoid by secreting prometastatic mediators, including Col6A1 and Lcn2 [154].

Recent studies have demonstrated that PDAC-derived exosomes play a critical role in pre-metastatic niche formation. Costa-Silva et al. [155] demonstrated that exosomes from the pancreatic primary tumor can be taken up by Kupffer cells, upregulating TGFβ secretion and fibronectin production in HSCs to form a fibrotic microenvironment in the liver, which can recruit macrophages by macrophage migration for immunosuppression. Additionally, a greater number of exosomes are found in stage I PDAC patients who later develop liver metastasis, indicating that exosomes could promote liver metastasis and may be a diagnostic marker [155]. In addition, CD44v6 can promote pancreatic cancer growth and metastasis through the MET and VEGFR2 pathway [156], and pre-metastatic niche formation induced by exosomes requires CD44v6 [157]. In another study of exosomes derived from human breast cancer and PDAC cell lines that metastasize to the lung, liver, or both, the expression patterns of integrins in exosomes were examined, and the results indicated that the integrins α6β4 and α6β1 are associated with lung metastasis, whereas integrin αvβ5 correlates with liver metastasis [158]. Moreover, exosome integrin uptake by resident cells (lung fibroblasts and epithelial cells, liver Kupffer cells) activates Src phosphorylation and pro-inflammatory S100 gene expression, which contributes to pre-metastatic niche formation [158].

Other immune cells can also facilitate pre-metastatic niche formation. For instance, TAMs can secrete granulin to activate HSC differentiation into myofibroblasts for fibrotic microenvironment formation in liver to support metastatic PDAC growth [159]. In addition, MDSCs and neutrophils participate in liver pre-metastasis niche formation via the CXCR2 pathway. CXCR2 is a G-protein-coupled receptor for human CXC chemokines to control neutrophil and MDSC migration [160–162]. Steele CW et al. demonstrated that CXCR2 or Ly6G^+^ (a marker of neutrophils and MDSCs) cell depletion and CXCR2 inhibition can abrogate metastasis in KPC (LSL-Kras^G12D/+^; LSL-Trp53^R172H/+^; Pdx1-Cre) mice by failing to establish a metastatic niche, while CXCR2 inhibition substantially enhances sensitivity to anti-PD1 immunotherapy and prolongs survival in KPC mice [163].

**Fig. 2:**
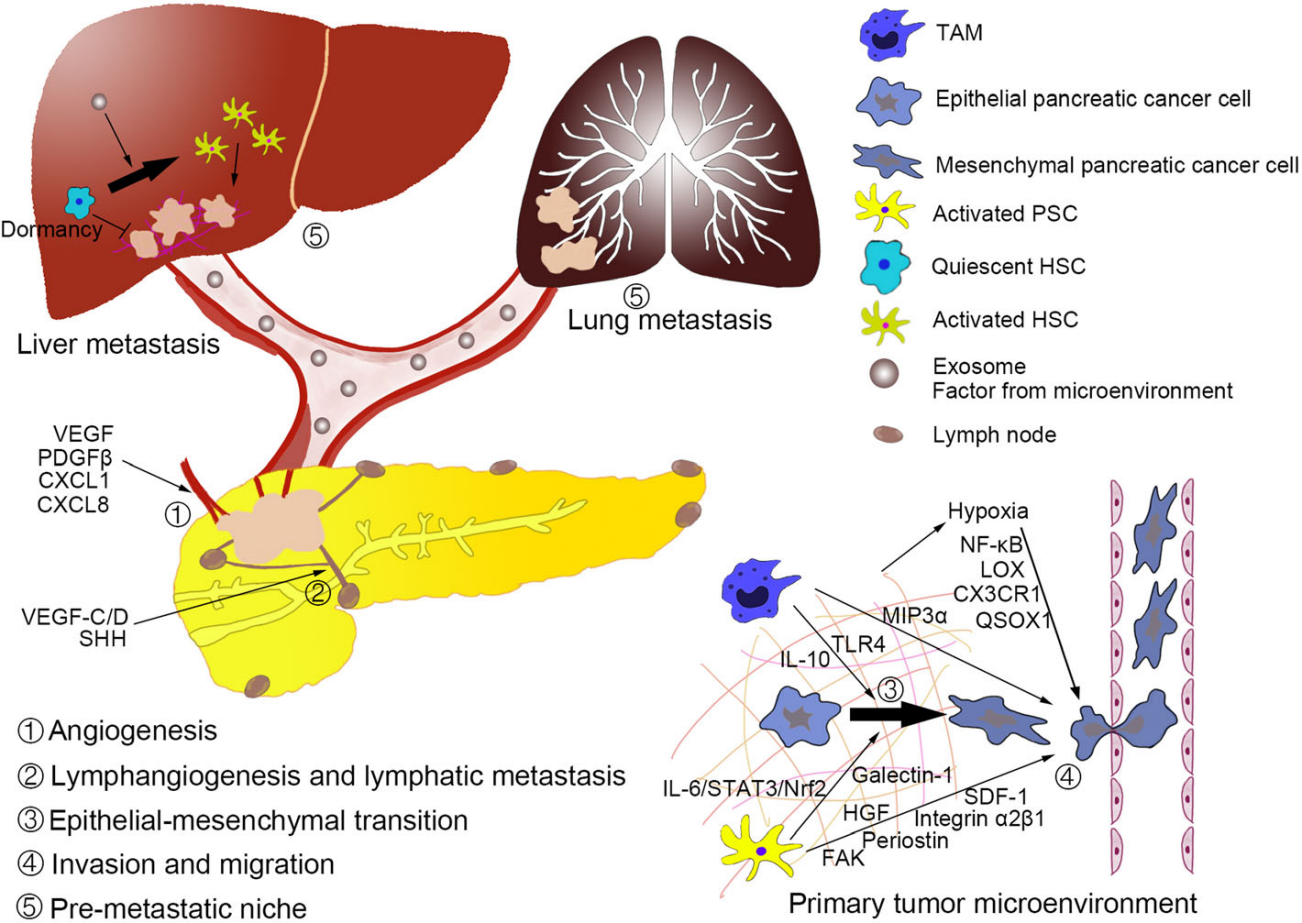
The pancreatic cancer microenvironment participates in metastasis. The pancreatic cancer microenvironment influences every step of metastasis via multiple signaling pathways. (1) The pancreatic cancer microenvironment can stimulate angiogenesis by cytokines to favor cancer cell survival and proliferation. (2) Molecules from the pancreatic cancer microenvironment can induce lymphangiogenesis to establish a pathway for lymphatic metastasis. (3) The pancreatic cancer microenvironment can facilitate the epithelial-mesenchymal transition to cause cancer cells to enter lymphatic vessel. (4) The pancreatic cancer microenvironment can play important roles in invasion and migration to facilitate metastasis. (5) Factors and exosomes derived from the pancreatic cancer microenvironment can induce pre-metastatic niche formation in liver and lung. These molecules or exosomes can activate hepatic stellate cells (HSCs) in liver for desmoplasia

Cancer dormancy at metastatic sites is responsible for the establishment of metastatic lesions and influenced by the (pre-) metastatic niche. In pancreatic cancer, downregulation of *KRAS* and *c-Myc* may be markers of dormant pancreatic cancer cells. Although *KARS* inhibition should be an effective strategy to treat pancreatic cancer, the rapid recurrence of primary and metastatic cancer following reactivation of *KRAS* supports the existence of dormant cancer cells in the animal model [164]. In addition, reactivation of *c-Myc* causes pancreatic cancer recurrence [165]. Lin et al. [165] demonstrated that *c-Myc*-negative tumor cells can express exogenous c-Myc, indicating that the dormant population is enriched by pancreatic CSCs. As mentioned before, the stemness of pancreatic CSCs is maintained by microenvironmental components such as TAMs and PSCs, indicating that TAMs and PSCs may participate in pancreatic cancer dormancy. Furthermore, Lenk et al. [166] found that liver sections of tumor-bearing KPC mice consisting of micrometastases displaying weakly proliferative and quiescent HSCs can mediate the quiescence-associated phenotype of pancreatic ductal epithelial cells, with a flattened cell morphology, Ki67-negativity and reduced proliferation, in coculture. This study showed that quiescent HSCs may induce pancreatic cancer dormancy in the liver metastatic niche, and a switch from quiescent to inflammatory activated HSC can enhance proliferation of pancreatic ductal epithelial cells (Fig. 2).

## Conclusions

Clinical studies have demonstrated that components within the pancreatic cancer microenvironment correlate with a poor prognosis of patients and can facilitate desmoplasia and immunosuppression or promote metastasis via numerous signaling pathways associated with the failure of immunotherapy, chemotherapy or radiation therapy. Pancreatic cancer cells can produce signaling molecules to recruit or activate stromal and immunosuppressive cells, such as Tregs, MDSCs, TAMs, and PSCs, even at a very early stage of pancreatic cancer development, to establish the microenvironment, which plays important roles in evolutionary and ecological processes in pancreatic cancer. The evolution of pancreatic cancer is divided into 3 steps: initiation, clonal expansion, and introduction to foreign microenvironments [167]. Based on the studies reviewed herein, we can infer that the selective pressures of the pancreatic cancer microenvironmental ecology can shape pancreatic cancer evolution. For example, in pancreatic cancer, TAMs can be recruited into the microenvironment to regulate the stemness of pancreatic CSCs for initiation, and pancreatic CSCs secrete factors such as IFNβ to stimulate TAMs for stemness maintenance; PSCs can secrete several factors to promote the growth of pancreatic cancer; factors/exosomes from the primary microenvironment can induce formation of the pre-metastatic niche in liver and lung; and the microenvironment in the metastatic niche can induce pancreatic cancer dormancy for recurrence.

Some factors secreted by cellular elements of the microenvironment are simultaneously involved in desmoplasia, immunosuppression, and metastasis. For example, TGFβ, IL-10 and VEGF, which are considered to be immunosuppressive cytokines secreted by pancreatic cancer cells, Tregs, and TAMs, can promote desmoplasia, angiogenesis/lymphangiogenesis, EMT, and formation of the pre-metastatic niche. STAT3, a star molecule in cancer progression, can activate other molecules involved in immunosuppression or metastasis. Thus, how are these factors regulated and what is their relationship to the 4 driver genes? Concerning the regulation of these factors, increasing knowledge of molecular pathogenesis has shown that these factors are epigenetically regulated by DNA methylation, histone modification or non-coding RNAs. For example, the hedgehog transcription factor Gli1 targets the epigenetic modifiers DNMT1 and DNMT3a, which are positive targets of oncogenic epigenetic pathways in pancreatic cancer [168]. MUC1, which participates in immunosuppression and the progression of pancreatic cancer, can be regulated by DNA methylation and histone H3 lysine 9 modification [169]. MiR-27a can activate the Ras/MAPK signaling pathway by inhibition of Sprouty2, the inhibitor of Ras/MAPK, in pancreatic cancer [170]. Moreover, exosomes, which are a hot topic in cancer research, contain non-coding RNAs and play a pivotal role in the liver metastasis of pancreatic cancer by altering the phenotype of the cells in the pre-metastatic niche. Therefore, understanding the regulation of these molecules will be important for identifying potential therapeutic targets.

A better understanding of the pathways in the tumor microenvironment during the metastasis of pancreatic cancer will facilitate a breakthrough in cancer immunotherapy studies and provide a rationale for clinical trials, which can contribute to improving the efficacy of therapy.

## Abbreviations

Akt: Protein kinase B
APC: Antigen-presenting cell
Arg1: Arginase 1
CAF: Cancer-associated fibroblast
CCL: CC chemokine ligand
Cdc42: Cell division control protein 42 homolog
*CDKN2A*: Cyclin-dependent kinase Inhibitor 2A
CEC: Circulating epithelial cell
c-Met: Tyrosine-protein kinase Met
CSC: Cancer stem cell
CTC: Circulating tumor cell
CTC: Circulating tumor cell
CTGF: Connective tissue growth factor
CTLA-4: Cytotoxic T-lymphocyte-associated protein 4
CXCL: CXC chemokine ligand
CXCR: CXC chemokine receptor
DC: Dendritic cell
ECM: Extracellular matrix
EGFR: Epidermal growth factor receptor
EpCAM: Epithelial cell adhesion molecule
Erk: Extracellular signal-regulated kinase
FAK: Focal adhesion kinase
Fas: First apoptosis signal
FGF2: Fibroblast growth factor 2
Foxp3: Forkheadbox protein 3
FPR2: Formyl peptide receptor 2
GM-CSF: Granulocyte macrophage colony-stimulating factor
HGF: Hepatocyte growth factor
HIF: Hypoxia inducible factor
HLA: Human leukocyte antigen
HSC: Hepatic stellate cell
ICAM-1: Intercellular adhesion molecular-1
IDO: Indoleamine 2,3-dioxygenase
IFN-γ: Interferon-γ
IL: Interleukin
iNOS: Inducible nitric oxide synthase
IP-10: Interferon gamma-induced protein 10
IPMN: Intraductal papillary mucinous neoplasm
*KRAS*: Kirsten rat sarcoma viral oncogene
LL: Leucine leucine
MAPK: Mitogen-activated protein kinase
MDSC: Myeloid-derived suppressor cell
MHC: Major histocompatibility complex
MIP: Macrophage inflammatory protein
MMP: Matrix metalloproteinase
MUC: Mucin
NF-κB: Nuclear factor kappa-light-chain-enhancer of activated B cells
NK: Nature killer
NLRP3: NACHT, LRR and PYD domains-containing protein 3
Nrf2: Nuclear factor erythroid 2
P2X7R: P2X purinoceptor 7 receptor
PD-1: Programmed cell death protein-1
PDAC: Pancreatic ductal adenocarcinoma
PDGF: Platelet-derived growth factor
PGE2: Prostaglandin E2
PI3K: Phosphoinositide 3-kinase
PMN-MDSC: Polymorphonuclear cells
PSC: Pancreatic stellate cell
RAF: Rapidly accelerated fibrosarcoma kinase
ROS: Reactive oxygen species
SDF-1: Stromal cell derived factor 1
SHH: Sonic hedgehog
shRNA: Short hairpin RNA
*SMAD4*: Mothers against decapentaplegic homolog 4
STAT3: Signal transducer and activator of transcription 3
TAM: Tumor-associated macrophage
TGFβ: Transforming growth factor-β
Th: T helper
TIL: Tumor infiltrating lymphocyte
TLR: Toll-like receptor
TRAIL: TNF-related apoptosis-inducing ligand
Treg: Regulatory T cell
VDR: Vitamin D receptor
VEGF: Vascular endothelial growth factor
α-SMA: α-Smooth muscle actin
